# Non-IgG4-Related Fibrosing Mediastinitis Diagnosed on Core Needle Biopsy and Treated with Steroids: A Case Study and Review of the Differential Diagnoses

**DOI:** 10.1177/10668969231219646

**Published:** 2024-01-17

**Authors:** Kseniia Malkova, Alyeesha B. Wilhelm, Hamza Uddin, Ikenna Okereke, Vidarshi Muthukumarana

**Affiliations:** 1 Department of Pathology, 12338 University of Texas Medical Branch, Galveston, TX, USA; 2 Department of Pathology, 6595 University of Pittsburg Medical Center, Pittsburgh, PA, USA; 3 Department of Surgery, 12338 University of Texas Medical Branch, Galveston, TX, USA; 4 Department of Surgery, 2971 Henry Ford Health System, Detroit, MI, USA

**Keywords:** fibrosing mediastinitis, core needle biopsy, IgG4-related disease, sclerosing mediastinitis

## Abstract

**Objectives:**

This study aimed to investigate the histological characteristics and treatment efficacy of non-immunoglobulin G4-related fibrosing mediastinitis and discuss differential diagnoses for this rare entity.

**Methods:**

We present a case study of non-immunoglobulin G4-related fibrosing mediastinitis diagnosed on core biopsy and treated with steroids. A total of four 18-gauge core needle biopsy specimens were obtained for surgical pathology. Analysis of the patient's medical history, radiological characteristics of fibrosing mediastinitis, histological features, immunohistochemistry results, the differential diagnosis and treatment efficacy of different types of fibrosing mediastinitis was performed.

**Results:**

This report describes a unique presentation of fibrosing mediastinitis (syncope and weight loss) that was concerning for malignancy. Histological, laboratory and radiographical studies confirmed the diagnosis of non-immunoglobulin G4-related fibrosing mediastinitis. The patient received corticosteroid treatment which showed marked improvement after 1 month of treatment.

**Conclusions:**

Fibrosing mediastinitis is an extremely uncommon entity with unknown pathogenesis, and it is more important to rule out malignancy and infection than to delineate between fibrosing mediastinitis and IgG4-related disease. In doing this, we may reasonably initiate a trial of corticosteroids which may prove beneficial, as in this patient. More studies on the pathogenesis of fibrosing mediastinitis are necessary to guide better directed treatments.

## Introduction

Fibrosing mediastinitis, also referred to as sclerosing mediastinitis, is an aggressive fibroinflammatory process with proliferation of locally invasive fibrous tissue within the mediastinum. It is a rare disorder and a difficult diagnosis to make as many malignancies may have a similar clinical presentation and fibrosing morphology (eg, lymphoma, carcinoma, and mesothelioma). Etiologies of fibrosing mediastinitis commonly include infections (notably histoplasmosis), medications, radiation therapy, and autoimmune conditions. Significant histopathologic overlap with IgG4-related disease makes the delineation between the two conditions difficult.^1^ The distinction between these disease processes is important, as immunosuppression is indicated for IgG4-related disease but not helpful for other causes of fibrosing mediastinitis. Additionally, immunosuppression could prove harmful in other conditions that have fibrosing morphology. Multiple biopsies, sometimes including minimally invasive approaches, can be required to establish a diagnosis of fibrosing mediastinitis. We report a unique case of non-IgG4-related fibrosing mediastinitis that mimicked malignancy on presentation was diagnosed on core needle biopsy and showed improvement after treatment with corticosteroids. A review of the differential diagnosis is included to aid in future diagnostic dilemmas.

## Case Report

**Figure 1.**
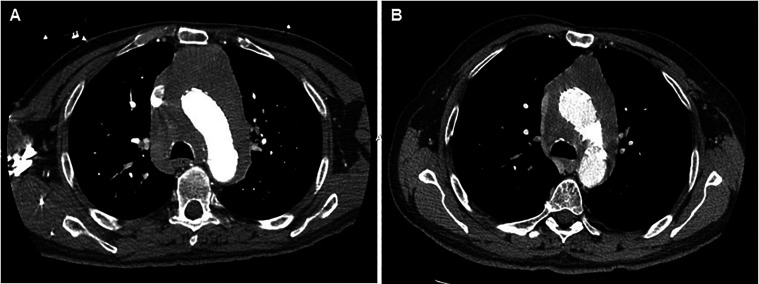
Ct angiogram of the chest showed a soft tissue infiltrative mass in the mediastinum replacing the mediastinal fat and encasing the aortic arch (A). The largest diameters at the level of the aortic arch were 11.0 × 10.0 cm. CT of the chest 1 month after treatment with steroids showed interval improvement in the size of the infiltrative lesion extending from the level of the arch vessels down to the aortic root with the largest diameters at the level of the aortic arch are 10.0 × 7.7 cm (B).

**Figure 2.**
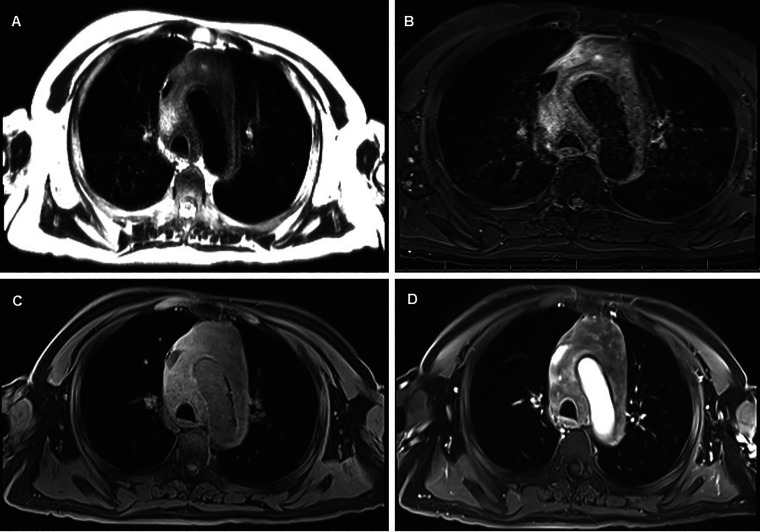
Magnetic resonance imaging (MRI) of the chest demonstrated an infiltrative mediastinal mass with low to intermediate signal intensity on T2 HASTE sequence (A), high signal intensity of T2 STIR sequence (B), intermediate to high signal intensity on T1-weigthed fat-saturated sequence (C), and irregular areas of enhancement on postcontrast sequence (D).

A 65-year-old Caucasian man with no significant past medical history presented with an unprovoked syncopal episode lasting 3–5 min without preceding chest pain, seizure-like activity or postictal state. He noted a 40-pound weight loss over the past four months. He had a 48 pack-year tobacco history but stopped 2 years prior to presentation. Physical exam was unremarkable. An electrocardiogram showed sinus rhythm and no abnormalities. He was worked up prior at an outside hospital for syncope. Computed tomography angiography (CTA) of the chest showed a large pericardial effusion and mediastinal heterogenous hypodense mass measuring 8.1 × 7.8 centimeters. The mass encased the aortic arch and was associated with lymphadenopathy in both hilar regions and his left supraclavicular area (Figure 1A). Magnetic resonance imaging of the chest also demonstrated an infiltrative mediastinal mass (Figure 2).

**Figure 3.**
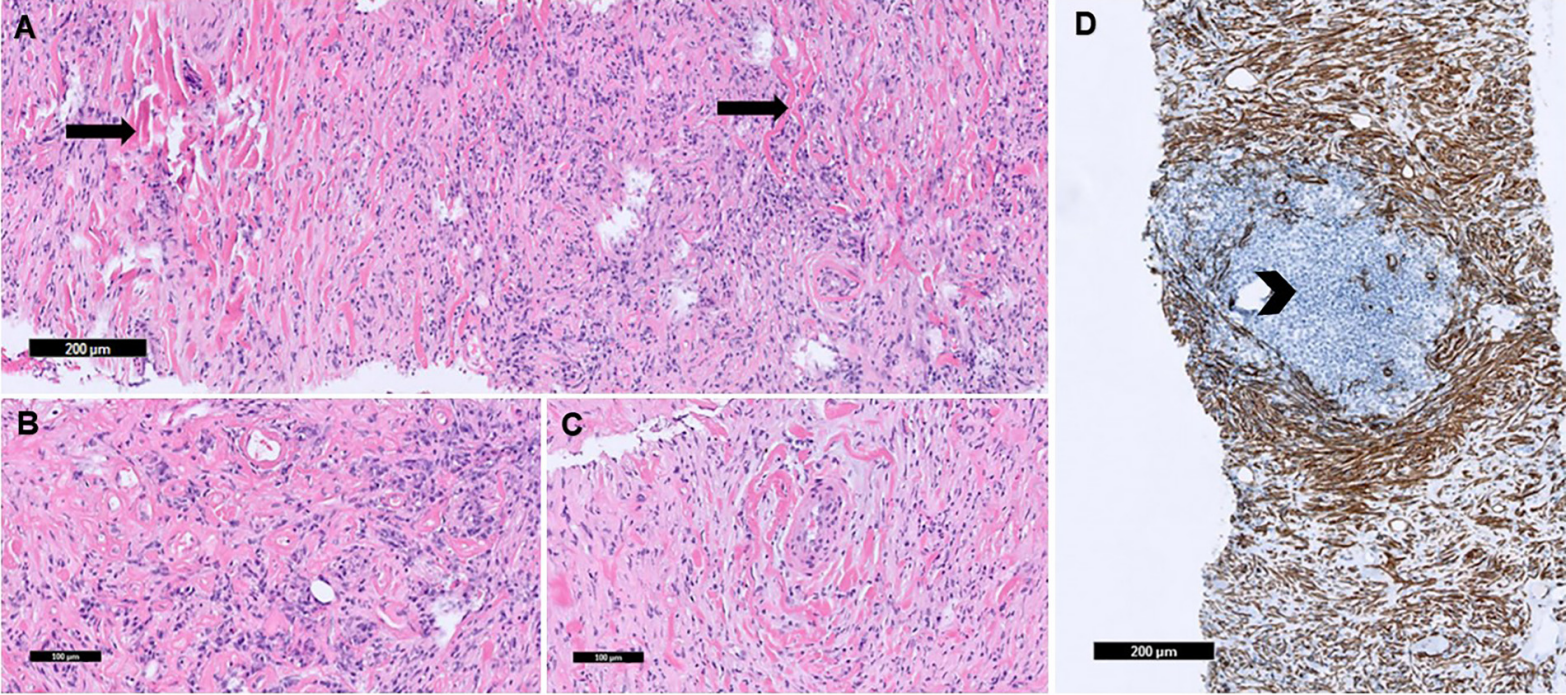
Tissue section showed fibrosis with keloid-type collagen (arrows) which circumscribe vessels (A), mixed inflammatory cells with plasma cell aggregates (B), and blood vessels surrounded by keloid-type collagen (C) (H&E stain). Smooth muscle actin (SMA) stain highlighted fibrosis with keloid-type collagen. Negative SMA staining highlights mixed inflammatory cells and few benign lymphoid follicles (arrowhead) (D).

Pericardiocentesis yielded 1 L of sanguineous fluid. An initial biopsy was conducted at an outside institution, but the sample was inadequate for diagnosis and a repeat biopsy was recommended. A repeat FNA and core needle biopsy by interventional radiology showed mixed inflammatory cells composed of plasma cells, lymphocytes and eosinophils in a background of dense fibrosis with keloid-type collagen and few benign lymphoid follicles (Figure 3). An immunohistochemistry (IHC) panel was performed (Table 1) and was non-supportive of a hematolymphoid malignancy, metastatic carcinoma, or infectious etiology.

**Table 1.** Immunohistochemical (IHC) and Special Stains.

PAX8	Negative
STAT-6	Negative
Keratin AE1/AE3	Negative
ALK	Negative
CD138	Highlighted scattered plasma cells
CD30	Negative
MUM1	Highlighted scattered plasma cells
PAX5	Highlighted rare B lymphocytes in follicles
Kappa	Polyclonal expression in plasma cells
Lambda	Polyclonal expression in plasma cells
Congo Red	Negative
KIT	Negative
AFB	Negative for acid fast bacilli
GMS	Negative for fungal organisms
SMA	Focally Positive
S100	Negative
Keratin 34 beta E12	Negative
CD21	Negative
IgG4/IgG ratio	0.1 (less than 0.4)
IgG4	Highlights up to 20 cells/HPF
CD3	Mixed positive T cells
CD20	Positive B cells
CD1a	Negative for Langerhans cell clusters

Paired box gene 8 (PAX8); signal transducer and activator of transcription 6 (STAT6); anticytokeratin monoclonal antibodies (AE1 and AE3); anaplastic lymphoma kinase 1 (ALK-1); plasma cell marker CD138; cell membrane protein of the tumor necrosis factor receptor family and a tumor marker CD30; multiple myeloma oncogene-1 (MUM1); paired box 5 (PAX5); acid fast bacilli (AFB); receprot tyrosine kinase (c-KIT); grocott methenamine silver stain (GMS); smooth muscle actin (SMA); high molecular weight keratin (keratin 34 beta E12); immunoglobulin G4 (IgG4); T-cell marker CD3; B-cell marker CD20; Langerhans cells and immature T cells marker CD1A.

Serum and tissue IgG4 were non-supportive of IgG4-related disease with an IgG4/IgG ratio of 0.1 by IHC, though there was polyclonal hypergammaglobulinemia. Overall histologic and clinical features supported a diagnosis of fibrosing mediastinitis. There was no laboratory evidence of fibrosing mediastinitis-associated infections such as histoplasmosis, tuberculosis, or syphilis. Laboratory values were significant for a positive ANA; however, extensive panel of other rheumatological serologies were negative. Of note, the patient denied parotid enlargement, oral and/or nasal ulcers, photosensitivity, rashes, joint pain or swelling, Raynaud's phenomenon or family history of autoimmune disease.

The patient was treated with a prednisone taper after consultation with rheumatology. Repeat CTA demonstrated almost complete resolution of the pericardial effusion, decrease in the size of the mass and reduction of the lymphadenopathy (Figure 1B).

## Discussion

Fibrosing mediastinitis is a rare benign diffuse disorder caused by proliferation of acellular collagen and fibrous tissue within the mediastinum.^2^ Fibrosing mediastinitis may represent a clinical-pathologic syndrome rather than a single disease.^2,3^ It is commonly associated with granulomatous or fungal infections, especially with histoplasmosis, however, only less than one percent lead to chronic disease.^4^ A case of *Wucheria bancrofti* parasitic infection causing chronic fibrosing mediastinitis also been published.^5^ The hypothesized pathophysiology of this disease is related to an immune response to fungal infection that have spread to the mediastinum causing an extensive fibrosis; the mechanism for noninfectious etiologies causing chronic fibrosing mediastinitis is unknown.^4^

Autoimmune disorders such as systemic lupus erythematosus, rheumatoid arthritis, antineutrophil cytoplasmic antibody-associated vasculitis, IgG4-related disease, Riedel's (fibrous) thyroiditis, and Behçet's syndrome were also previously described as the cause of fibrosing mediastinitis.^6^ There has been a case reported of fibrosing mediastinitis mimicking sarcoidosis.^7^ Fibrosing mediastinitis can be also associated with malignant soft tissue or hematological neoplasms, or can be caused by radiation or drug therapy (methysergide).^8^

Fibrous mediastinitis is typically asymptomatic until the fibrosis causes compression or obstruction of mediastinal organs. The symptoms may differ depending on anatomic structures involved. The superior vena cava is most compromised causing superior vena cava syndrome. The patient may also have chest pain, shortness of breath, wheezing, cough, dysphagia or hemoptysis; patients may also have syncopal episodes due to great vessels or heart compression in most severe cases or signs of pulmonary hypertension.^9^

Our patient was admitted for syncope and weight loss; the imaging studies revealed mediastinal mass and large pericardial effusion. The differential for fibrosing lesions identified on FNA and biopsy in the mediastinum is large. Our differential diagnosis mainly included malignant neoplasms (thymoma, lymphomas, lung cancer, metastatic carcinomas), infectious etiologies and benign or malignant mesenchymal tumors. Morphological assessment and IHC play significant role in differential diagnosis.

Sclerosing thymoma composed of hyalinized, fibrosclerotic stroma that expands into septa, perivascular spaces, and the tumor periphery. It was argued against because sclerosing mediastinitis shows hyalinized collagen with only foci of inflammatory aggregates rather than the dual cell population and lacks focal areas of spindle epithelial cells like in thymomas.^10^

Primary mediastinal (thymic) large B cell lymphoma (sclerosing diffuse large B-cell lymphoma) demonstrates diffuse infiltration of mediastinal soft tissue and surrounding structures with atypical B cells therefore the CD20 and or CD79a markers should be positive.^11^ Nodular sclerosis variant of Hodgkin disease histologically represents sclerotic lesions with nodules of eosinophils and histiocytes admixed with lymphocytes and plasma cells. Reed-Sternberg cells would express positivity for CD15+, CD30+, and MUM1, and weakly positive for PAX5.^12^ Sclerosing mediastinitis lacks the population of large atypical B cells. Rare lymphoproliferative differentials include Erdheim-Chester Disease^13^ and Castleman disease.^14^

Tumors with spindle cell and fibrous morphology are also important group for differential diagnosis. Diffuse desmoplastic malignant mesothelioma is a hypercellular spindle cell tumor with dense paucicellular hyalinized collagen on a background and characteristic immunohistochemical staining for keratin.^15^ Aggressive low-grade peripheral sheath tumor and neurofibroma cells show spindle cell morphology and are positive for S100 and SOX10 and negative for MUC4.^16^ In solitary fibrous tumor spindle cells have homogeneous to ovoid nuclei and an indistinct cytoplasm and are positive for CD34 and STAT6, and negative for cytokeratins, S100, EMA, muscle markers and melanoma-associated markers.^17^ Pulmonary and mediastinal synovial sarcomas^16,18^ and low-grade fibromyxoid sarcomas^19^ are extremely rare, and only few cases were published in the literature.

Extra abdominal form of deep (aggressive) fibromatosis can involve thorax. Histologically, it is spindle cell neoplasm with ill define borders. IHC usually demonstrates positive staining for vimentin, β-catenin; and negative staining for S100, CD34, CD99, Bcl2.^20^

Also, it is important to exclude carcinomas from other anatomic cites metastasizing to mediastinum and initiating reactive fibroinflammatory response. It is also possible that this biopsy represents a nonspecific reactive fibroinflammatory change occurring adjacent to a neoplasm which was not sampled. Therefore, re-biopsy is suggested in challenging cases where clinical suspicion for malignancy is high.^21^

Based on exclusion all other etiologies, IgG4-related sclerosing mediastinitis was considered as our main differential. Radiological findings typically demonstrate periaortic and paravertebral masses and patients usually respond well to glucocorticoid and immunosuppressant treatment.^22^ IgG4-related fibrosing mediastinitis should have more than two of the following major features: (a) dense lymphoplasmacytic infiltration with greater than 40% IgG4+/IgG + plasma cells, (b) storiform fibrosis; and (c) obliterative phlebitis.28 In 2012, Umehara et al proposed a comprehensive diagnostic criteria: (a) Organ swelling, mass or nodular lesions, or organ dysfunction; (b) a serum IgG4 concentration > 135 mg/dl; and (c) histopathological findings of >10 IgG4 cells/high power field and an IgG4þ/IgGþ cell ratio > 40%.^23^

Importantly, all the differential diagnoses described above may exhibit areas with substantial fibrosis, therefore, biopsy samples obtained with a percutaneous needle technique may yield insufficient tissue to rule out malignancy. Open biopsies with extensive sampling are frequently required to establish a definitive diagnosis.^2^ Our case showed that in some cases the diagnosis of non-IgG4-related fibrosing mediastinitis can be made without extensive sampling; however, the correlation with laboratory studies, radiographic features, and IHC may be very helpful to make the correct diagnosis, even given limited tissue sampling.

IgG4-related fibrosing mediastinitis, like other IgG4-related disease, can respond to glucocorticoids, typically with symptomatic improvement and size reductions in the tumor or affected organ. Inoue et al reported the case of IgG4-related fibrosing mediastinitis with high levels of serum IgG4 that showed remission of the symptoms after steroid therapy and suggested serum IgG4 levels as a good indicator for steroid therapy in these cases.^24^

Studies have shown that in the setting of idiopathic fibrosing mediastinitis (non-IgG4 fibrosing mediastinitis with no known etiology), therapeutic success using systemic glucocorticoids and other immunosuppressant agents is exceptionally rare.^1^ In one review, only a single case of non-IgG4-related fibrosing mediastinitis showed response to steroid; improvements were short-lived and the patient subsequently died of progressive pulmonary artery compression.^3^ Therefore, it is crucial to differentiate IgG4-related sclerosing mediastinitis from similar lesions in order to select the proper treatment.^25^

Our case showed no evidence of IgG4-relaed disease by serology or IHC. Our tissue sample was sufficient to show adequate IgG positivity without IgG4 positivity, and serum levels of IgG4 were not supportive of IgG4-related fibrosing mediastinitis. Additionally, there was no histological or laboratory evidence of infection with histoplasmosis, though a historical infection could not be completely ruled out. In a case with similar laboratory findings, Kagan et al report that, unlike IgG4-related disease in which glucocorticoids are effective, there is no documented effective treatment for non-IgG4-related fibrosing mediastinitis and the case was fatal.^26^ Another case of non-IgG4-related fibrosing mediastinitis reported by Oka et al reports that they were unsure as to whether or not they should treat with steroids.^27^ Our patient, however, was diagnosed as non-IgG4 fibrosing mediastinitis and treatment with glucocorticoids has proven effective, as least thus far.

## Conclusion

This case describes a unique presentation of fibrosing mediastinitis with syncope and weight loss that was concerning for malignancy. Diagnosis of spindle cell and/or sclerosing tumors in the mediastinum is dependent on histopathology—the interpretation of which may be difficult and is often precluded by limited tissue sampling. Confidence in adequate tissue sampling from sclerosing lesions can be achieved with thorough laboratory and radiographical correlation. In cases with histopathological features of IgG4-related disease that lack serology and IHC for IgG4, it is more important to rule out malignancy and infection than to delineate between fibrosing mediastinitis and IgG4-related disease. In doing this, we may reasonably initiate a trial of corticosteroids which may prove beneficial, as in this patient. More studies on the pathogenesis of fibrosing mediastinitis are necessary to guide better directed treatments.
